# Single‐Cell and Spatial Analysis Reveal the Differences Between Left‐Sided and Right‐Sided Colorectal Cancer

**DOI:** 10.1111/cpr.70260

**Published:** 2026-07-14

**Authors:** Zongnai Zhang, Jiaqi Xu, Yingchao Wu, Tao Liu, Junling Zhang, Mai Zhou, Jie Qiao, Heng Pan, Xin Wang

**Affiliations:** ^1^ Department of General Surgery Peking University First Hospital, Peking University Beijing China; ^2^ Department of General Surgery Civil Aviation General Hospital Beijing China; ^3^ State Key Laboratory of Female Fertility Promotion, Center for Reproductive Medicine, Department of Obstetrics and Gynecology Peking University Third Hospital Beijing China; ^4^ Key Laboratory of Assisted Reproduction (Peking University), Ministry of Education Beijing China; ^5^ Beijing Key Laboratory of Collaborative Innovation in Frontier Technologies for Population Quality Beijing China; ^6^ National Clinical Research Center for Obstetrics and Gynecology (Peking University Third Hospital) Beijing China

**Keywords:** colorectal cancer, tertiary lymphoid structure, tumour laterality, tumour microenvironment

## Abstract

Colorectal cancer (CRC) exhibits heterogeneity based on tumour laterality, with right‐sided tumours demonstrating distinct genetic, immunogenic and clinical behaviours compared to left‐sided counterparts. In this study, we conducted single‐cell RNA sequencing and spatial transcriptomics to dissect the distinct molecular landscapes of left‐ and right‐sided CRC. We identified enhanced germinal centre B cell infiltration and CXCL13^+^ T cell enrichment, features linked to adaptive immune priming and better immunotherapy responsiveness. Right‐sided CRC epithelial cells exhibited proliferative and immunogenic phenotypes, marked by upregulated immune‐related pathways and chemokine‐driven interactions with lymphocytes. Spatial analysis revealed organized tertiary lymphoid structure‐immune microenvironment crosstalk in right‐sided tumours, mediated by CCL19/21‐CCR7 signalling. In contrast, left‐sided CRC tumours displayed stromal‐epithelial interactions favouring angiogenesis and metabolic reprogramming. Our findings established a laterality‐specific immune microenvironment in CRC, providing insights for precise therapeutic strategies of left‐ and right‐sided CRC.

## Graphical abstract

Single‐cell and spatial analysis revealed distinct CRC microenvironments by tumour laterality. Right‐sided tumours showed enhanced germinal centre B cells, CXCL13+ T cells, and organized TLS‐immune crosstalk via CCL19/21‐CCR7, suggesting better immunotherapy response. Left‐sided tumours exhibited stromal‐epithelial interactions promoting angiogenesis and metabolism.

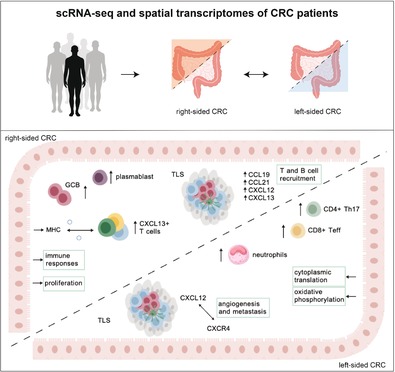

## 1 Introduction

Colorectal cancer (CRC) is one of the most prevalent malignancies and remains a major cause of cancer‐related mortality globally [1]. It encompasses a heterogeneous group of cancers that can arise from distinct anatomical locations of the large intestine: the left‐sided (descending colon, sigmoid colon, and rectum) and right‐sided (cecum, ascending colon, and transverse colon). The left‐sided and right‐sided CRC exhibit marked differences in embryological origin, clinical outcomes, genetic landscapes, and responses to therapy, underscoring the importance of understanding their distinct biological characteristics [1, 2, 3, 4, 5, 6].

Right‐sided CRC tumours are often characterized by frequent mutations in the DNA mismatch repair pathway, leading to a higher incidence of mismatch repair deficiency (MMRd) and a greater prevalence of microsatellite instability‐high (MSI‐high) [6, 7]. On the contrary, left‐sided CRC tumours are typically microsatellite stable (MSS), with a lower incidence of mismatch repair deficiency (MMRp) and different chromosomal instability patterns (CIN‐high) [6, 7].

In addition to genetic discrepancies, CRC exhibits a large dynamic range of immune responses, harbouring striking differences between MMRd and MMRp subtypes: MMRd tumours are more immunogenic, harbouring activated T cell infiltrates, and exhibiting stronger responses to immunotherapies compared to their MMRp counterparts [8, 9, 10, 11, 12, 13, 14, 15]. Notably, emerging evidence suggests that these immunogenic disparities may be further amplified by the presence of tertiary lymphoid structures (TLS)—ectopic and organized lymphocyte aggregates that orchestrate adaptive immune responses within tumour microenvironments [16]. TLS are often found in chronic inflammatory sites, including cancer, and are primarily composed of B cells, T cells, and dendritic cells, which may support the recruitment of lymphocytes and maintain an immune‐responsive microenvironment [17]. The presence of TLS has been reported in various cancer types, including CRC, non–small cell lung cancer, ovarian cancer, and melanoma [18, 19, 20, 21, 22]. Moreover, several recent studies have demonstrated that TLS are associated with improved prognosis and enhanced responses to immune checkpoint blockade (ICB) therapies [17, 23, 24, 25, 26].

Despite these insights, a comprehensive understanding of the molecular and cellular distinctiveness of left‐sided and right‐sided CRC remains insufficiently explored. By employing single‐cell RNA‐seq (scRNA‐seq) in conjunction with spatial transcriptome (ST), we aim to detail the cellular heterogeneity within left‐ and right‐sided CRC to enhance our understanding of CRC pathogenesis and the underlying biological disparities that might dictate differential disease behaviour and treatments. We identified robust immunological disparities between the two subtypes, including enhanced germinal centre B cell infiltration, CXCL13‐driven T cell coordination, and TLS maturation in right‐sided CRC. Additionally, we uncovered proliferative and immunogenic epithelial subpopulations in right‐sided CRC, alongside spatially resolved communication networks that sustained immune activation. These findings provide a multi‐dimensional atlas of CRC laterality, linking cellular heterogeneity to differential therapeutic vulnerabilities and offering actionable insights for precision immunotherapy strategies.

## 2 Results

### 2.1 A Single‐Cell Transcriptomic Atlas of Left‐Sided and Right‐Sided CRC

**FIGURE 1:**
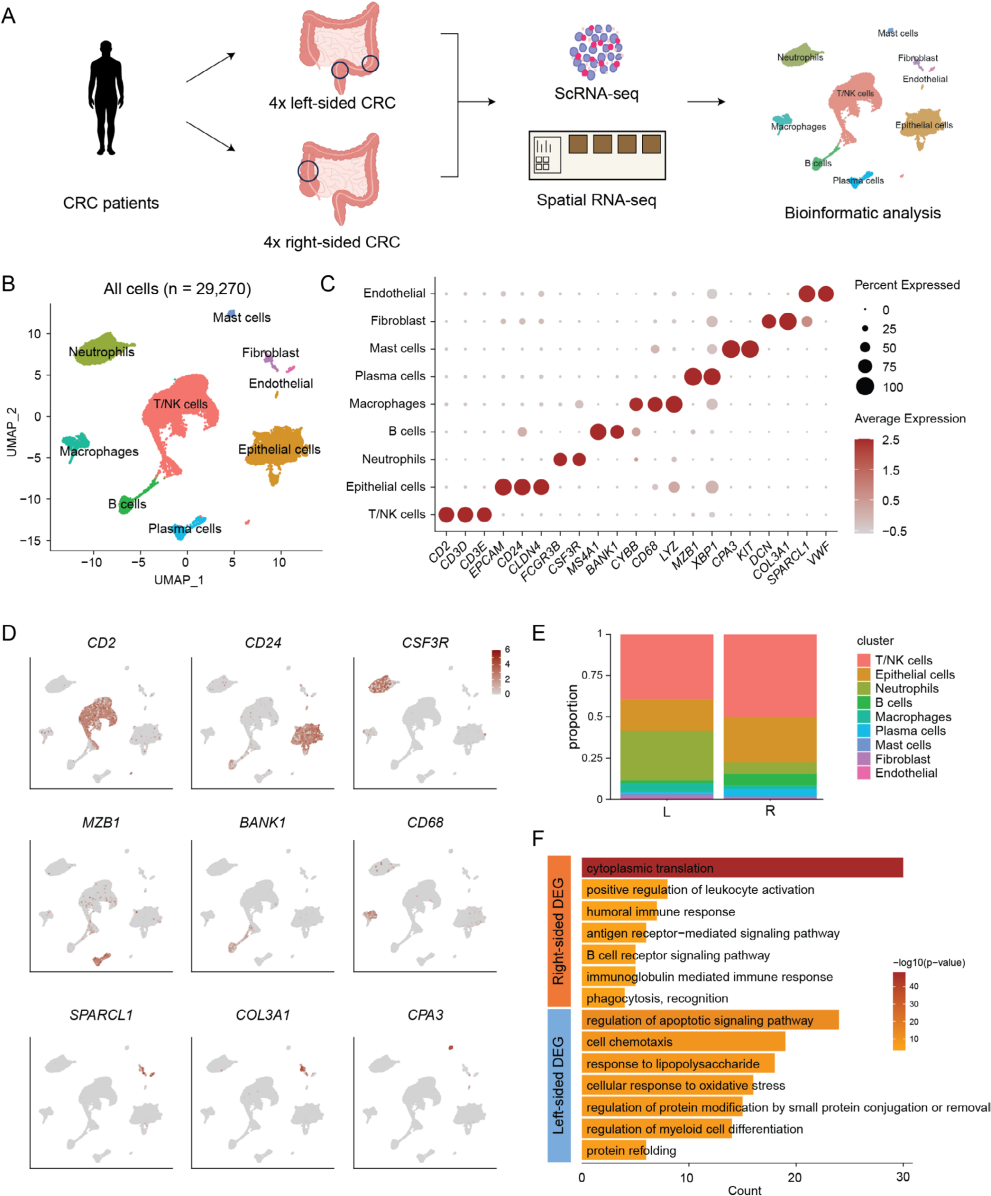
Single‐cell transcriptomic atlas of left‐sided and right‐sided CRC. (A) Schematic diagram of the study design. scRNA‐seq was processed using the 10× Chromium 3′ v3 kit (10× Genomics) and sequenced on a NovaSeq 6000 platform. (B) UMAP by cell clusters. (C) Dot plot depicting the relative expression of representative known marker genes for clusters. (D) Expression levels of representative known markers illustrated in UMAP plots. (E) Bar plot illustrating the distinct proportions of major cell types in left‐sided versus right‐sided CRC. (F) Enriched GO pathways in left‐sided and right‐sided CRC.

To elucidate the differences between tumour tissues from left‐sided and right‐sided CRC, we generated single‐cell transcriptomes from four samples of each type (Figure 1A; Table S1). After rigorous quality control and filtering, 29,270 high‐quality single‐cell transcriptomes were retained for subsequent analysis (Figure 1B, Section 4). To correct batch effects across samples, we integrated scRNA‐seq data using the Harmony algorithm [27] (Figure S1A). We then employed the Harmony‐corrected principal components to construct a unified UMAP embedding space, performed graph‐based clustering, and annotated each cluster with its differentially expressed gene (DEG) signatures (Figure 1B; Figure S1B).

Cells were classified into nine major cell types, including T/NK cells (*CD2*, *CD3D* and *CD3E*), epithelial cells (*EPCAM*, *CD24* and *CLDN4*), neutrophils (*FCGR3B* and *CSF3R*), B cells (*MS4A1* and *BANK1*), macrophages (*CYBB*, *CD68* and *LYZ*), plasma cells (*MZB1* and *XBP1*), mast cells (*CPA3* and *KIT*), fibroblasts (*DCN* and *COL3A1*), and endothelial cells (*SPARCL1* and *VWF*) (Figure 1B–D; Figure S1C). Although plasma cells derive from the B‐cell lineage, their markedly distinct transcriptomic signature led us to analyse them as a separate population. Despite the presence of all major cell types in both left‐ and right‐sided CRC, their proportions varied, possibly reflecting differences between these subtypes (Figure 1E). Notably, the right‐sided CRC showed a trend toward a larger proportion of T/NK cells, B cells, and plasma cells compared to the left‐sided CRC (Figure 1E).

To systematically dissect the molecular distinctions between left‐ and right‐sided CRC, we first identified global DEGs across all cell types. Pathway enrichment analysis revealed that right‐sided CRC exhibited pronounced activation of immune‐related processes, including positive regulation of leukocyte activation, humoral immune response, antigen receptor‐mediated signalling pathway, and B cell receptor signalling pathway, whereas left‐sided CRC was enriched in regulation of apoptotic signalling pathway, cell chemotaxis, and cellular response to oxidative stress (Figure 1F). Consistent with prior research, our results also suggested a more immunologically active microenvironment in right‐sided CRC [6].

### 2.2 Remodelling of the T Cell Compartment in Left‐Sided and Right‐Sided CRC

**FIGURE 2:**
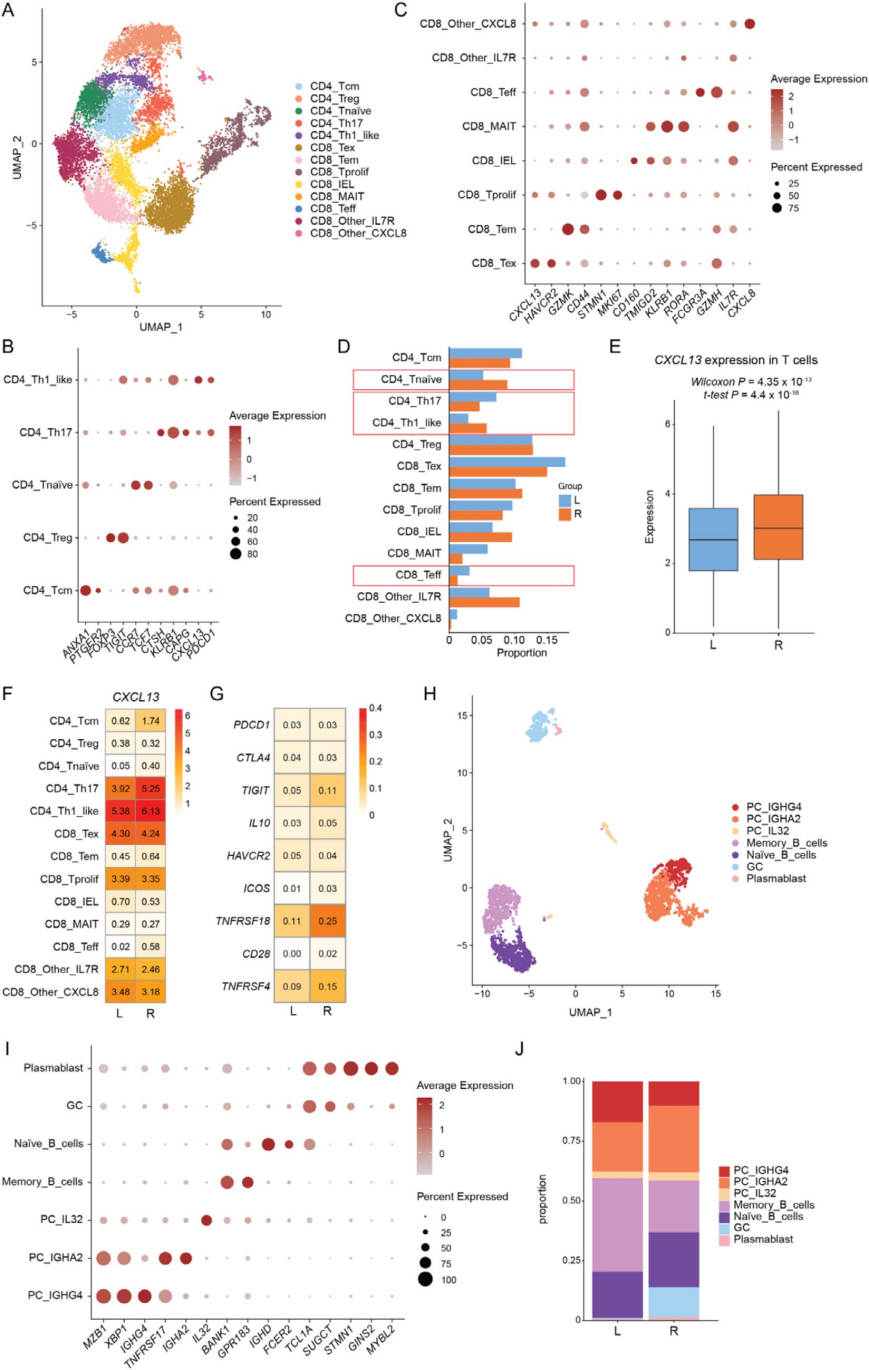
More lymphocyte infiltration in right‐sided CRC. (A) UMAP of T cells by sub‐clusters. (B) Dot plot depicting the relative expression of representative known markers for each CD4^+^ T cell subcluster. (C) Dot plot depicting the relative expression of representative known markers for each CD8^+^ T cell subcluster. (D) The proportion of T cell subclusters in left‐sided and right‐sided CRC. (E) The *CXCL13* expression is higher in right‐sided CRC. Statistical significance was assessed using the Wilcoxon rank‐sum test and two‐tailed *t*‐test. (F) Heatmap of log‐transformed average expression of *CXCL13* in each T cell subcluster. (G) Heatmap of log‐transformed average expression of immune checkpoint‐related genes in B cells from left‐sided and right‐sided CRC. (H) UMAP of B cells by subclusters. (I) Dot plot depicting the relative expression of representative known markers for each B cell subcluster. (J) Bar plot illustrating the distinct proportions of B cell subclusters in left‐sided versus right‐sided CRC.

Given the prominence of immune pathways in right‐sided CRC, we focused on the remodelling of the T cell compartment. Unsupervised subclustering of T cells resolved CD4^+^ and CD8^+^ T cells (Figure S2A). Specifically, CD4^+^ T cells were further divided into CD4^+^ central memory T cells (*ANXA1* and *PTGER2*), CD4^+^ regulatory T cells (*FOXP3* and *TIGIT*), CD4^+^ naïve T cells (*CCR7* and *TCF7*), CD4^+^ T helper 17 cells (*CTSH*, *KLRB1* and *CAPG*), and CD4^+^ T helper 1‐like cells (*CXCL13* and *PDCD1*) (Figure 2A,B; Figure S2B). CD8^+^ T cells were categorized into exhausted CD8^+^ T cells (*CXCL13* and *HAVCR2*), CD8^+^ T effector memory cells (*GZMK* and *CD44*), proliferating T cells (*STMN1* and *MKI67*), CD8^+^ intraepithelial lymphocytes (*CD160* and *TMIGD2*), mucosal associated invariant T cells (*KLRB1* and *RORA*), CD8^+^ effector T cells (*FCGR3A* and *GZMH*), and two CD8^+^ clusters with *IL7R* and *CXCL8* expression (Figure 2A,C; Figure S2B).

By focusing on the differences between left‐ and right‐sided CRC, we identified a moderate divergence in CD4^+^ T cell polarization between tumour laterality. Consistent with previous research [28], a larger fraction of CD4^+^ naïve T cells was from right‐sided CRC, corroborating their role in maintaining adaptive immune plasticity (Figure 2D). Notably, Th1‐like cells were enriched in the right‐sided microenvironment, whereas left‐sided tumours exhibited Th17‐skewed differentiation (Figure 2D). Intriguingly, this dichotomy mirrors the Th1/Th17 polarization observed between microsatellite MSI‐H and MSS CRC [29]. Given the observation that the combination of PD‐1 and CTLA‐4 immune blockade directly triggered a Th1‐like response, and the involvement of Th1‐like cells was implicated in response to anti‐CTLA4 therapy in melanoma [30, 31], we hypothesized that the enrichment of Th1‐like cells in right‐sided CRC might contribute to its better response to immunotherapies. Future studies with larger cohorts and functional assays will be required to test this hypothesis. We also uncovered a dominance of CD8^+^ effector T cells in left‐sided tumours (Figure 2D). This observation aligned with a recent report of an accumulation of CD8^+^ effector T cells in left‐sided CRC [28], yet it seemingly contrasts with the paradigm of right‐sided tumours harbouring stronger anti‐tumour immunity, which remains to be further explored. These subset proportion analyses, derived from a limited number of samples, should be considered hypothesis‐generating and warrant confirmation in larger cohorts.

The chemokine CXCL13 has gained increasing attention in various cancers, including CRC, as a hallmark of tumour‐reactive T cells and potentially as a biomarker for favourable outcomes following immunotherapy [32, 33, 34]. CXCL13 is also critical for TLS formation and B cell recruitment [35, 36]. The enrichment of CXCL13^+^ T cells was identified in MMRd tumours compared to MMRp tumours [7]. Notably, right‐sided CRC exhibited globally significantly elevated *CXCL13* expression across CXCL13^+^ T cells (Figure 2E, Wilcoxon rank‐sum test *p* = 4.35 × 10^−13^; *t*‐test *p* = 4.4 × 10^−16^), with various T cell subclusters exhibiting elevated average *CXCL13* expression levels (Figure 2F). This T cell CXCL13 enrichment aligned with reports linking CD8^+^CXCL13^+^ cell abundance to favourable PD‐1 blockade outcomes [15], suggesting that right‐sided CRC's CXCL13 signature may preconfigure an immunotherapy‐responsive niche, likely through increasing B cell infiltration, enhancing collaboration between T cells and B cells, fostering TLS maturation, and sustaining clonal expansion of tumour‐specific T cells.

Collectively, our T cell analysis delineated a right‐sided CRC‐specific immunophenotype characterized by Th1 polarization and CXCL13‐driven immune coordination, features that may synergistically underlie the reported clinical advantage of right‐sided tumours in immune ICB therapies. However, given the absence of immunotherapy outcome data in our cohort, these functional implications remain to be further investigated.

### 2.3 B Cell Functional Diversification in Left‐Sided and Right‐Sided CRC

Right‐sided CRC exhibited enhanced B cell and plasma cell infiltration (Figure 1E). Notably, the expression levels of immune‐checkpoint‐related genes (*TIGIT*, *TNFRSF4*, etc.) were higher in right‐sided CRC, which might account for its better response to immunotherapies (Figure 2G). Subclustering B and plasma cells resolved eight distinctive subclusters, including Immunoglobulin G (IgG^+^) plasma cells (*MZB1*, *XBP1*, and *IGHG4*), Immunoglobulin A (IgA^+^) plasma cells (*MZB1*, *XBP1*, *TNFRSF17* and *IGHA2*), IL32^+^ plasma cell (*MZB1*, *XBP1*, and *IL32*), memory B cells (*BANK1* and *GPR183*), naïve B cells (*IGHD* and *FCER2*), germinal centre B cells (*TCL1A* and *SUGCT*), and plasmablasts (*STMN1*, *GINS2*, and *MYBL2*) (Figure 2H,I; Figure S2C). Notably, germinal centre B cells (GCB) were almost exclusively enriched in right‐sided CRC, with similar findings documented in the ascending tumour (Figure 2J) [37]. GCB drive antibody diversification through somatic hypermutation and class‐switch recombination, enabling the production of high‐affinity antibodies against antigens, including tumours [38]. As vital components of mature TLS, GCB are linked to better prognosis and enhanced response to immunotherapies in various human cancers [17, 23, 24, 25, 26, 39].

The marked enrichment of GCB and elevated CXCL13 levels in T cells of right‐sided CRC suggested an immune hub where GCB might orchestrate humoral and cellular crosstalk. This niche might support the generation of tumour‐specific antibodies or might directly induce complement‐mediated cytotoxicity. These findings provided a potential mechanistic foundation for the enhanced immunotherapy response of right‐sided CRC, highlighting a compelling direction for further investigation.

### 2.4 Enrichment of Proliferative and Immunogenic Epithelial Cells in Right‐Sided CRC

**FIGURE 3:**
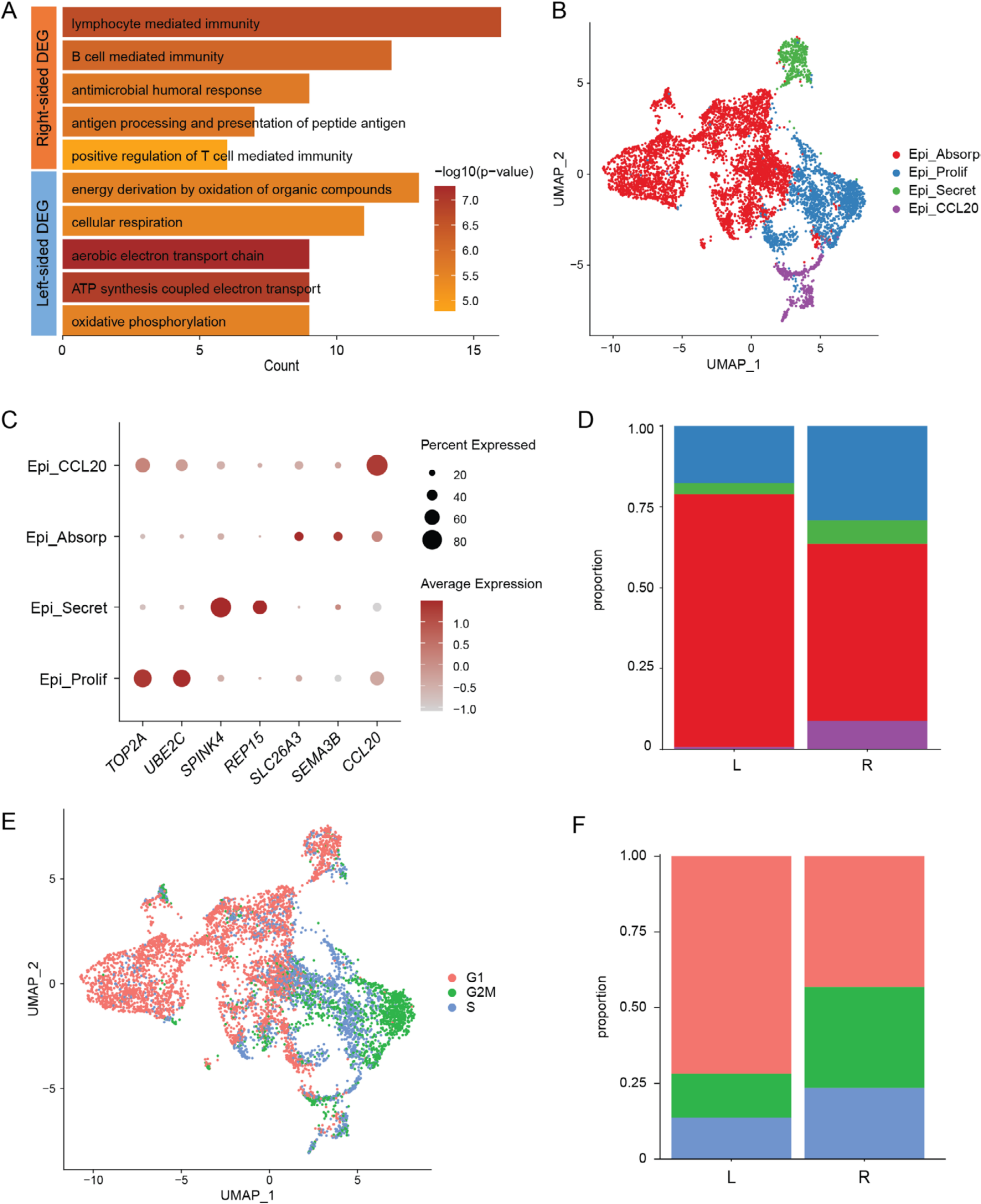
More proliferative and immunogenic epithelial cells are enriched in right‐sided CRC. (A) Enriched GO pathways in epithelial cells from left‐ and right‐sided CRC. (B) UMAP by subclusters. (C) Dot plot depicting the relative expression of representative known markers for each subcluster. (D) Different proportions of subclusters shown in bar plot from left‐ and right‐sided CRC. (E) UMAP by cell cycle assignment based on signature genes. (F) Enrichment of G2/M and S phase cells in right‐sided CRC.

Transcriptomic profiling of epithelial cells revealed profound laterality‐associated functional divergence. Pathway enrichment analysis demonstrated that epithelial cells in right‐sided CRC were significantly enriched for immune‐related processes, including lymphocyte‐mediated immunity, humoral immune response, B cell‐mediated immunity, positive regulation of T cell‐mediated immunity, and antigen processing and presentation of endogenous antigen (Figure 3A). In contrast, left‐sided tumours were enriched for cytoplasmic translation, oxidative phosphorylation, and ATP synthesis coupled electron transport (Figure 3A). These findings corroborated the observation that right‐sided CRC exhibits a more immunogenic profile and suggested that epithelial cells in right‐sided CRC may contribute to immune responses.

To further investigate the differences between epithelial cells derived from left‐ and right‐sided CRC, we re‐clustered epithelial cells into four subclusters, including proliferative epithelial cells (*TOP2A* and *UBE2C*), secretory epithelial cells (*SPINK4* and *REP15*), absorptive epithelial cells (*SLC26A3* and *SEMA3B*), and CCL20^+^ epithelial cells (*CCL20*) (Figure 3B,C; Figure S3). Notably, right‐sided CRC harboured the subcluster co‐expressing immune markers alongside canonical epithelial markers (Figure 3D), potentially representing epithelial cells that engage in the crosstalk with filtering lymphocytes.

Coupled with this immune‐primed state, right‐sided CRC displayed heightened proliferative activity. Cell cycle phase scoring revealed a higher proportion of cells in S/G2M phases in right‐sided CRC compared to left‐sided CRC (Figure 3E,F). This coexistence of immune activation and proliferation suggested a unique ecosystem in the right‐sided CRC.

### 2.5 Distinct Intercellular Communications in Left‐Sided and Right‐Sided CRC

**FIGURE 4:**
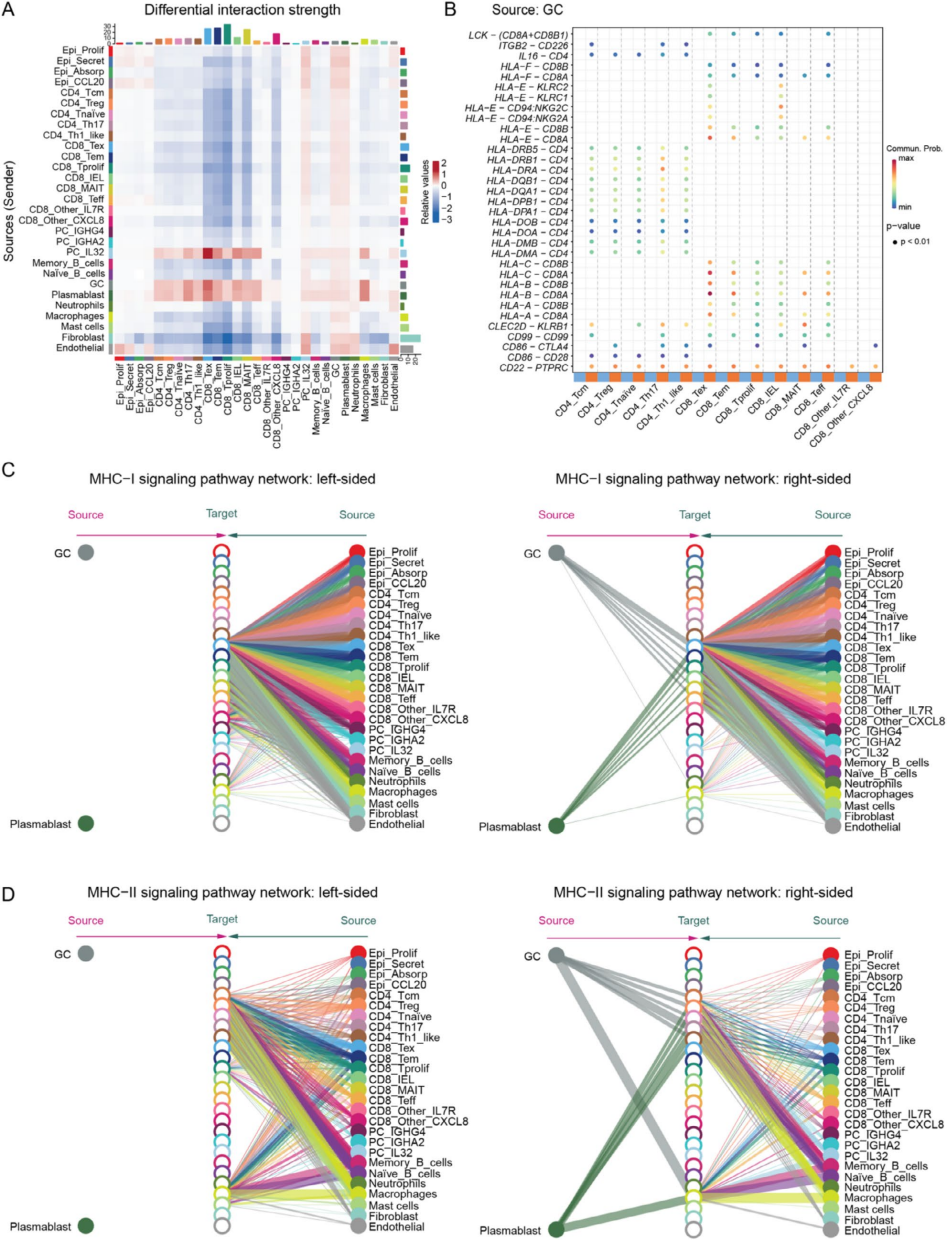
Intercellular communications between subclusters. (A) Heatmap showing the differential strength of interactions. Blue, enriched in the left‐sided CRC. Red, enriched in right‐sided CRC. (B) Bubble heatmap showing communication probability between clusters for ligand‐receptor pairs using GCB as source. (C) Different MHCI signalling in left‐ and right‐sided CRC. (D) Different MHCII signalling in left‐ and right‐sided CRC.

To unravel how cell subclusters interact in the tumour microenvironment, we employed CellChat to map ligand‐receptor networks [40]. Global cell–cell interaction analysis revealed distinct communication strengths between clusters of left‐ and right‐sided CRC (Figure 4A; Figure S4A,B). While left‐sided tumours exhibited higher overall interaction strength, right‐sided CRC displayed enhanced interactions between GCB, plasmablasts, and other subclusters (Figure 4A; Figure S4B), forming a coordinated immune activation environment.

In right‐sided CRC, MHCI–CD8 pairs and MHCII–CD4 pairs were significantly enriched between GCB and CD4^+^ and CD8^+^ T cell subsets (Figure 4B–D). MHCI molecules present peptides derived from self or foreign antigens to CD8^+^ T cells, and their loss in cancer cells leads to immunotherapy resistance and worse prognosis [41, 42, 43, 44]. MHC class II expression, typically restricted to antigen‐presenting cells, enables direct activation of CD4^+^ helper T cells. In glioblastoma, the enhanced expression of MHCII was shown to facilitate CD4^+^ T cell priming, driving anti‐tumour responses and is necessary for prolonged response to treatments [45]. The co‐enrichment of MHCI/II in right‐sided GCB might suggest it as an unconventional immune orchestrator, which enhanced adaptive immunity and hence potentially explained its improved responses to immunotherapies. These findings offered critical insights into the molecular difference between left‐ and right‐sided CRC, which are valuable for developing targeted therapies that address the unique characteristics of these tumours.

### 2.6 Detection of Para‐Tumour Tertiary Lymphoid Structures

**FIGURE 5:**
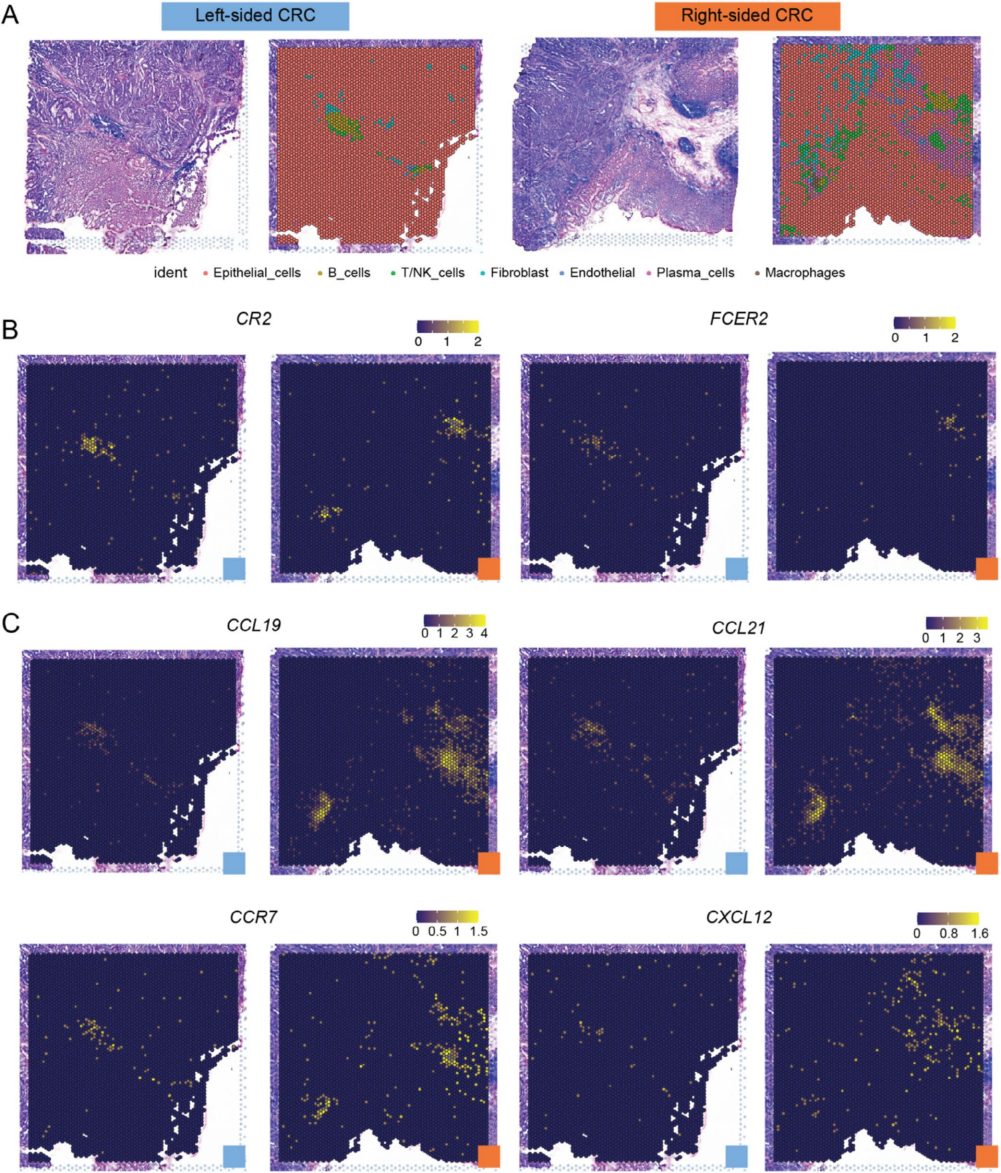
Para tumour TLS‐like structures revealed by spatial transcriptomics. (A) H&E staining of tissue sections and clustering of ST spots in left‐ and right‐sided CRC. (B) Spatial feature plots of gene expression of *CR2* and *FCER2*. (C) Spatial feature plots of gene expression of chemokines.

In CRC, TLS is often found below the invasive margin of tumours [46]. While scRNA‐seq revealed enrichment of GCB and CXCL13^+^ T cells in right‐sided CRC, this approach lacks spatial resolution and is therefore limited in dissecting TLS architecture. To address this, we performed spatial transcriptomics (ST) on tissue samples collected from the boundaries between left‐ and right‐sided CRC and adjacent normal tissue (L4 and R4) (Table S1). The tumour and the para‐tumour area were discriminated by haematoxylin and eosin (H&E) staining (Figure 5A). ST spots were annotated via scRNA‐seq projection into epithelial cells, B cells, T/NK cells, fibroblasts, endothelial cells, plasma cells, and macrophages (Figure 5A).

In the para‐tumour regions of both left‐ and right‐sided CRC, we identified cellular aggregates resembling TLS, characterized by colocalization of B cells and T cells expressing germinal centre markers (*CR2* and *FCER2*) (Figure 5A,B; Figure S5A). In the tumour nests we examined, these structures were not observed, where epithelial and endothelial cells predominated with sparse immune infiltration (Figure S5B).

Notably, TLS‐like niches in right‐sided CRC displayed elevated expression of chemokines *CCL19*, *CCL21*, *CCR7*, and *CXCL12* compared to left‐sided counterparts (Figure 5C). Chemokines CCL19, CCL21, CXCL12, and CXCL13 play important roles in facilitating the formation of TLS [16, 47, 48]. CCL19 was shown to promote TLS formation in CRC liver metastasis [49]. Additionally, CCL19 and CCL21 interact with CCR7 to attract T cells and facilitate the formation of the T‐cell zone in TLS in cancers [32].

### 2.7 Spatiotemporal Crosstalk Between TLS and the Tumour Environment in Left‐Sided and Right‐Sided CRC

**FIGURE 6:**
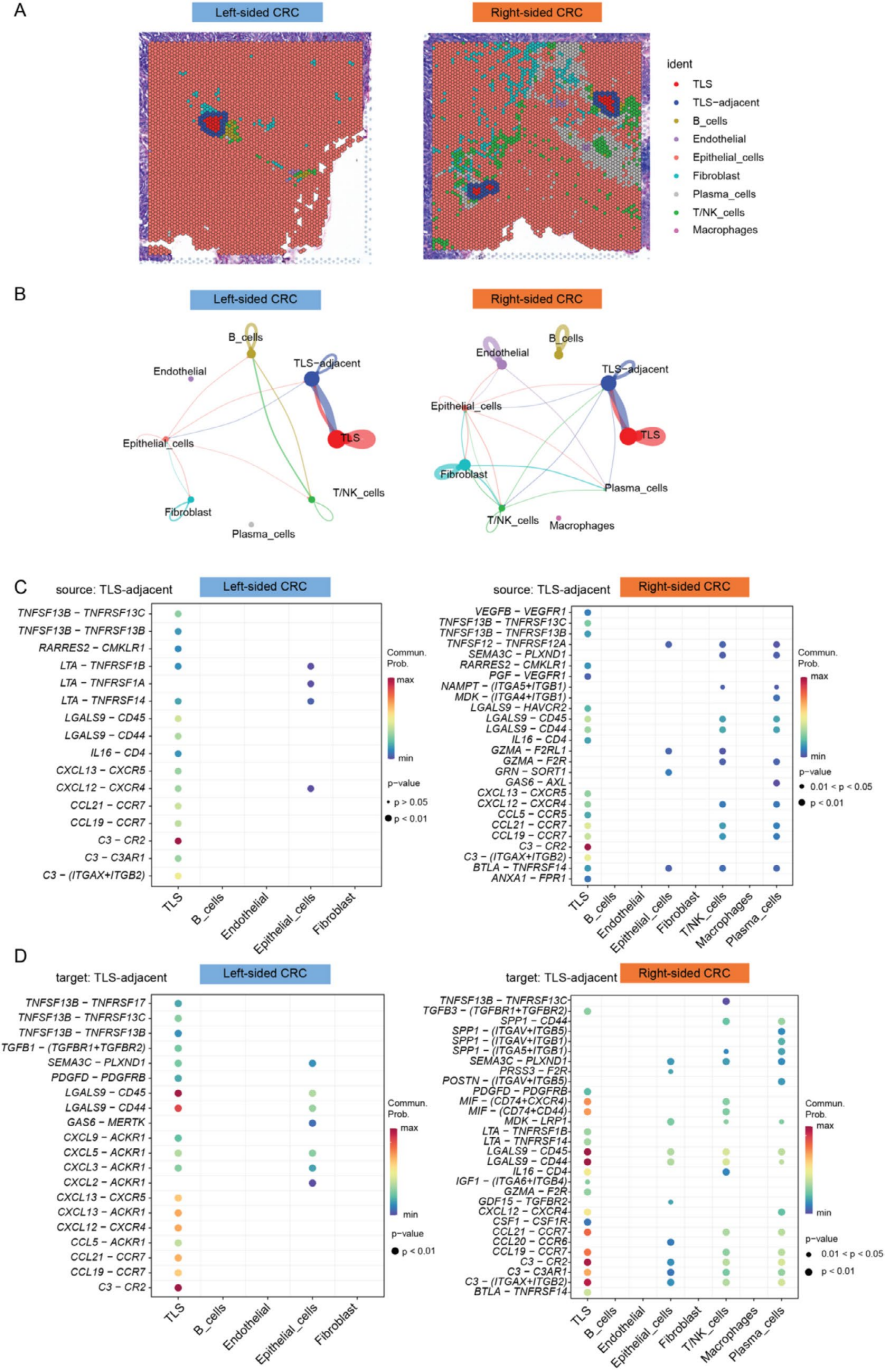
Spatially resolved TLS‐related communications. (A) Clustering of ST spots in left‐ and right‐sided CRC with TLS regions and TLS‐adjacent regions labelled. (B) Interactions between clusters in left‐ and right‐sided CRC. (C) Bubble heatmap showing communication probability between clusters for ligand‐receptor pairs using TLS‐adjacent cluster as source. (D) Bubble heatmap showing communication probability between clusters for ligand‐receptor pairs using TLS‐adjacent cluster as target. Plasma cells (B‐lineage derived) are shown separately to reflect their distinct spatial and functional roles in the TLS microenvironment.

To delineate how TLS interfaces with the tumour microenvironment, we first mapped TLS boundaries using spatial scoring of B cell, T cell, follicular dendritic cell, and germinal centre markers, defining TLS‐core regions and TLS‐adjacent zones (Figure 6A; Figure S6A, Section 4). While recognizing that plasma cells are derived from the B‐cell lineage, they were treated as separate nodes in this analysis because they formed distinct main clusters based on transcriptomic profiles and also showed different spatial localizations in the tissue (Figures 1B,C, 5A). CellChat analysis of spatially resolved ligand‐receptor networks revealed enhanced TLS‐TLS adjacent‐microenvironment communication in right‐sided CRC (Figure 6B). While TLS‐core regions primarily interacted with TLS‐adjacent regions (Figure S6B), TLS‐adjacent regions exhibited various interactions with TLS and surrounding cells, likely being important for TLS formation and maturation (Figure 6C,D).

In left‐sided CRC, the TLS‐adjacent exhibited several interactions with epithelial cells, including CXCL12‐CXCR4, which was linked to angiogenesis and metastasis in cancer [50, 51] (Figure 6C). In contrast, right‐sided CRC displayed various immune‐centric interactions (Figure 6C,D). TLS‐adjacent zones engaged plasma cells, T cells, and epithelial cells through CCL21‐CCR7, CCL19‐CCR7, and CCL20‐CCR6 signalling (Figure 6C,D). These chemokine axes guide the distribution of lymphocytes that express receptors CCR7 and CXCR5, thereby allowing the formation of T cell and B cell zones, which is a prerequisite of TLS formation [16, 48, 52]. These interactions likely fostered the maturation of TLS, creating a self‐reinforcing niche that amplified anti‐tumour immunity and underlied the enhanced immunotherapy responsiveness of right‐sided CRC. Therefore, the spatially resolved TLS‐related communication landscape underscored a fundamental dichotomy between left‐ and right‐sided CRC.

## 3 Discussion

Our study delineates fundamental immunological and spatial distinctions between left‐ and right‐sided CRC. The multi‐dimensional atlas presented here demonstrates that tumour laterality dictates distinct principles of immune microenvironment organization, with profound implications for precision immunotherapy. At the core of this divergence lies the differential capacity to establish and maintain functional TLS; organized immune hubs play vital roles in adaptive anti‐tumour immunity.

The right‐sided tumours behave more immunogenically by the presence of germinal centre B cells, higher expression of CXCL13^+^ in T cells, elevated immune‐related pathways in epithelial cells, which might amplify lymphocyte recruitment and enhanced TLS crosstalk with the immune microenvironment. These features align with established immune‐rich signatures and offer a potential explanation for the clinical observations of enhanced immunotherapy responses in right‐sided CRC [6]. The enrichment of CXCL13^+^ T cells and CCL19/21‐CCR7 signalling in right‐sided CRC underscores the role of chemokine networks in TLS formation and lymphocyte recruitment.

While prior studies linked TLS to improved prognosis [17, 23, 24, 25, 26], spatially resolved communication networks further illuminate how anatomical location shapes functional specialization. Notably, MHC I/II expression in germinal centre B cells highlights their non‐canonical antigen‐presenting functions, a finding warranting mechanistic validation. This structured immune education system may explain the superior immunotherapy responses observed in right‐sided CRC. In contrast, left‐sided tumours exhibit enhanced pro‐angiogenic and metabolic pathways.

Methodologically, our work demonstrates the necessity of spatial context in interpreting single‐cell data. While scRNA‐seq identified GCB enrichment, only spatial analysis revealed their precise organization within TLS architecture and communication with adjacent niches. This multi‐modal approach should become standard for deciphering complex tumour ecosystems.

Our study has several limitations that should be considered, the most significant being the sample size of both the scRNA‐seq and ST cohorts. We acknowledge that our cohorts of eight patients limit the broad statistical inferences that can be drawn, necessitating validation in larger, multi‐centre cohorts to ensure generalizability across populations with diverse genetic and environmental backgrounds. Regarding TLS identification, we acknowledge that the 55 μm spot diameter of the Visium platform cannot fully resolve the intricate microstructure of these structures. To mitigate this limitation, we implemented a multi‐faceted approach integrating scRNA‐seq data for cell‐type deconvolution, applying curated TLS marker gene signatures, and corroborating these with histological features from matched tissue images. Nevertheless, the precise anatomical details of TLS architecture remain beyond the current resolution, and our definition of TLS regions, while validated against histopathological features, remains heuristic. Future studies employing standardized, automated approaches with higher‐resolution spatial technologies will be essential to validate and refine our observations. Furthermore, while our CellChat inferences were consistent across probability thresholds and supported by spatial co‐localization in available samples, they remain predictive and require functional validation. More broadly, we acknowledge that our study is primarily descriptive, delineating associations rather than establishing causal mechanisms. The laterality‐specific features we identified provide a foundation of testable hypotheses that warrant mechanistic investigation through in vivo models and functional perturbation experiments. More importantly, our study lacks direct immunotherapy outcome data; thus, the association between right‐sided immune features and therapy response is speculative and highlights a critical need for prospective clinical validation.

Nevertheless, by delineating the cellular and spatial heterogeneity associated with CRC laterality, our study unveils a comprehensive landscape of molecular distinctions. This work is primarily descriptive and hypothesis‐generating, aiming to provide a systematic atlas of laterality‐specific features for the research community. For example, the enrichment of germinal centre B cells and CXCL13^+^ T cells in right‐sided CRC raises testable questions about their functional roles in TLS maturation and adaptive immunity. From a translational perspective, the immune active landscape of right‐sided tumours suggests that these patients may be particularly suitable for immunotherapies, a hypothesis that could be explored in laterality‐stratified cohorts. Conversely, the angiogenic and metabolic programmes enriched in left‐sided tumours point toward alternative therapeutic avenues, such as antiangiogenic or metabolic interventions. We believe this atlas will serve as a valuable resource to guide and prioritize future studies aimed at validating these laterality‐specific features and translating them into clinical applications.

## 4 Method

### 4.1 Clinical Sample Collection

Adjacent normal mucosa and tumour tissues were collected from CRC patients with informed written consent, and under approval of local medical ethics from Peking University First Hospital. In total, eight CRC patients aged from 34 years old to 77 years old were included. The cohort comprised two females and six males. All patients were *BRAF* wild type. None of the patients received chemoradiotherapy before surgery. Detailed information about the patients is listed in Table S1.

### 4.2 Single‐Cell RNA Library Preparation and Sequencing

Freshly prepared cell suspensions were performed immediately according to the manufacturer's protocol of 10× Chromium 3′ v3 kit (10× Genomics, Pleasanton, CA). The library was prepared, and sequencing was performed on a NovaSeq 6000 platform. The sequencing data were mapped to the human reference sequence (GRCh38) by cellranger (v6.0.0). The raw gene expression matrix from each sample was aggregated and converted into a Seurat object via the Seurat R package (v4.3.0.1, RRID: SCR_007322). Cells with < 200 genes or > 25% mitochondrial genes were filtered out. To remove potential doublets, we applied DoubletFinder [53] (v2.0.3) with default parameters. Ambient RNA contamination was eliminated using DecontX [54] (v1.14.2), and cells with contamination > 0.2 were excluded from downstream analysis. In total, we got 29,270 high‐quality cells for further analysis, including 11,173 cells from left‐sided CRC, and 18,097 cells from right‐sided CRC. To correct the batch effect, we used Harmony [27] to correct principal components. The harmonized PCA space (top 50 PCs) was used for UMAP embedding. We used the FindClusters function to cluster the cells. Feature expression plots were generated with the FeaturePlot function in Seurat.

### 4.3 Unsupervised Cell Clustering and Annotation

According to the DEGs of each lineage, cells were divided into T/NK cells, epithelial cells, neutrophils, B cells, macrophages, plasma cells, mast cells, fibroblasts and endothelial cells. To identify subclusters within these major cell types, a second‐round UMAP reduction was performed. DEGs of each subset were identified using the FindAllMarkers function implemented in Seurat.

### 4.4 Identification of DEGs and Pathway Enrichment

DEGs were identified by the FindMarkers function of the R Seurat package with the LR test using the sample as a latent variable. Genes with log2FC > 0.5 were considered as DEGs. GO enrichment was performed via the enrichGO function in ClusterProfiler (v4.6.2, RRID: SCR_016884).

### 4.5 Cell Cycle

Cell cycle phase was assigned using Seurat's CellCycleScoring with Seurat's built‐in S/M phase markers.

### 4.6 Cell–Cell Communications

CellChat (v1.6.1, RRID: SCR_021946) was employed to infer ligand‐receptor interactions [40]. We used the netVisual_diffInteraction function to draw a circle plot showing differential cell–cell communication between left‐ and right‐sided CRC. We used the compareInteractions function to compare the number and strength of cell–cell interactions in left‐ and right‐sided CRC. We used the netVisual_bubble function to visualize significant ligand‐receptor pairs between cell subclusters. We used the netVisual_aggregate function to visualize selected signalling pathways. While acknowledging their shared B‐cell lineage and noting their distinct spatial distributions, B cells and plasma cells were treated as separate groups to capture their specific ligand‐receptor interactions.

### 4.7 Spatial Transcriptome

The capture of gene expression information for ST slides was performed by the Visium Spatial platform of 10× Genomics with the default protocol. Raw sequencing reads of ST were quality checked and mapped by Space Ranger (v2.0.0, RRID: SCR_025848). The gene‐spot matrices generated after ST data processing were analysed with the Seurat package (v4.3.0.1, RRID: SCR_007322) in R. Spots were normalized by SCTransform. Spots were annotated by projecting the cell cluster annotation of scRNA‐seq onto ST via the FindTransferAnchors and the TransferData function. Signature scoring derived from ST signatures was performed with the AddModuleScore function with default parameters in Seurat. Spatial feature expression plots were generated with the SpatialFeaturePlot function in Seurat.

### 4.8 Defining TLS and TLS‐Adjacent Region

TLS regions were defined by an integrative spatial analysis pipeline. Signature scores thresholds of *MS4A1*, *CD3E*, *CR2*, and *FCER2* were established, with spatial spots exceeding a normalized score threshold of 0.8 classified as positively enriched for the corresponding cellular component.

Candidate TLS regions were initially defined as spatial coordinates containing at least one positively enriched cell type. To refine TLS boundaries, spatial neighbourhood analysis was performed by calculating the mean signature score of adjacent spots. Coordinates achieving a neighbourhood consensus threshold (mean positive markers ≥ 2/3 of adjacent spots) were retained as definitive TLS regions. TLS‐adjacent regions were operationally defined as all spatial spots localized within a 2‐row and 3‐column radius from TLS boundaries in the coordinate system, ensuring proximity while avoiding direct overlap with TLS cores. These computationally defined TLS regions were consistent with the morphological features of TLS observed on H&E‐stained sections.

### 4.9 Statistics

For scRNA‐seq data, DEGs were identified using the FindMarkers function in Seurat with the likelihood‐ratio test, incorporating sample identity as a latent variable to account for inter‐individual variability. Genes with absolute log_2_ fold change > 0.5 were considered differentially expressed. Pathway enrichment was assessed using ClusterProfiler with the hypergeometric test, and *p* values were adjusted for multiple testing using the Benjamini–Hochberg method. For comparing *CXCL13* expression scores of left‐ and right‐sided CRC, the Wilcoxon rank‐sum test and two‐tailed unpaired Student's *t*‐test were employed.

## Author Contributions

Z.Z., X.W., H.P. and J.Q. designed the study. Z.Z. and Y.W. collected clinical samples. J.X. performed bioinformatics analysis. J.X. and Z.Z. prepared figures and wrote the manuscript. H.P. and X.W. critically revised the manuscript. T.L., J.Z. and M.Z. helped with sample collection and manuscript revision. All authors have actively participated in data interpretation and have given their approval for the final version.

## Funding

This work was supported by the National Key Research and Development Program of China, 2023YFC2705901; National Natural Science Foundation of China, 82372860, 82401943; China Postdoctoral Science Foundation, GZB20240044.

## Ethics Statement

This study was approved by the local medical ethics committee from Peking University First Hospital.

## Conflicts of Interest

The authors declare no conflicts of interest.

## Supporting information


**Table S1:** Patient information.
**Figure S1:** Single‐cell transcriptomic atlas of left‐sided and right‐sided CRC. (A) UMAP by samples before and after batch effect removal. (B) UMAP by cell clusters of left‐sided and right‐sided CRC. (C) Expression levels of representative known markers illustrated in UMAP plots.
**Figure S2:** T cell and B cell subclusters. (A) Dot plot of CD4 and CD8A expression in subclusters. (B) Expression levels of representative known markers in CD4+ and CD8+ T cells are illustrated in UMAP plots. (C) Expression levels of representative known markers in B cells are illustrated in UMAP plots.
**Figure S3:** Epithelial subclusters. Expression levels of representative known markers illustrated in UMAP plots.
**Figure S4:** Intercellular communications between sub‐clusters. (A) Number of interactions and strength of interactions in left‐sided and right‐sided CRC. (B) Circo plots showing the differential number of interactions and the differential strength of interactions. Blue, enriched in the left‐sided CRC. Red, enriched in right‐sided CRC.
**Figure S5:** Spatial transcriptomics revealed gene expression and clustering. (A) Spatial feature plots of gene expression of CD3E and MS4A1. (B) Clustering of ST spots in left‐ and right‐sided CRC nests.
**Figure S6:** Spatial transcriptomics revealing identification of cell types and interactions. (A) Identification of T/B cells, follicular dendrite cells and germinal centres based on add module scores. (B) Bubble heatmap showing the communication probability between clusters for ligand‐receptor pairs using the TLS cluster as source.

## Data Availability

The data that support the findings of this study are openly available in the Gene Sequence Archive for Human database at https://ngdc.cncb.ac.cn/gsub/submit/bioproject/subPRO087900/overview, reference number PRJCA059956.
