# Supplementary material for: Single‐Cell and Spatial Analysis Reveal the Differences Between Left‐Sided and Right‐Sided Colorectal Cancer
Source: Cell Prolif. 2026 Jul 14:e70260. Online ahead of print. doi: 10.1111/cpr.70260 (PMC13366197; doi:10.1111/cpr.70260)
Supplement: Supplementary file 1 — Table S1: Patient information. Figure S1: Single‐cell transcriptomic atlas of left‐sided and right‐sided CRC. (A) UMAP by samples before and after batch effect removal. (B) UMAP by cell clusters of left‐sided and right‐sided CRC. (C) Expression levels of representative known markers illustrated in UMAP plots. Figure S2: T cell and B cell subclusters. (A) Dot plot of CD4 and CD8A expression in subclusters. (B) Expression levels of representative known markers in CD4+ and CD8+ T cells are illustrated in UMAP plots. (C) Expression levels of representative known markers in B cells are illustrated in UMAP plots. Figure S3: Epithelial subclusters. Expression levels of representative known markers illustrated in UMAP plots. Figure S4: Intercellular communications between sub‐clusters. (A) Number of interactions and strength of interactions in left‐sided and right‐sided CRC. (B) Circo plots showing the differential number of interactions and the differential strength of interactions. Blue, enriched in the left‐sided CRC. Red, enriched in right‐sided CRC. Figure S5: Spatial transcriptomics revealed gene expression and clustering. (A) Spatial feature plots of gene expression of CD3E and MS4A1. (B) Clustering of ST spots in left‐ and right‐sided CRC nests. Figure S6: Spatial transcriptomics revealing identification of cell types and interactions. (A) Identification of T/B cells, follicular dendrite cells and germinal centres based on add module scores. (B) Bubble heatmap showing the communication probability between clusters for ligand‐receptor pairs using the TLS cluster as source. [file CPR-9999-e70260-s001.docx]

**Supplemental information for:**

**Single-cell and spatial analysis reveal the differences between left-sided and right-sided colorectal cancer**

Zongnai Zhang, Jiaqi Xu, Yingchao Wu, Tao Liu, Junling Zhang, Mai Zhou, Jie Yan, Jie Qiao, Heng Pan, Xin Wang

**This pdf file contains:**

Figure S1-S6

Table S1

**
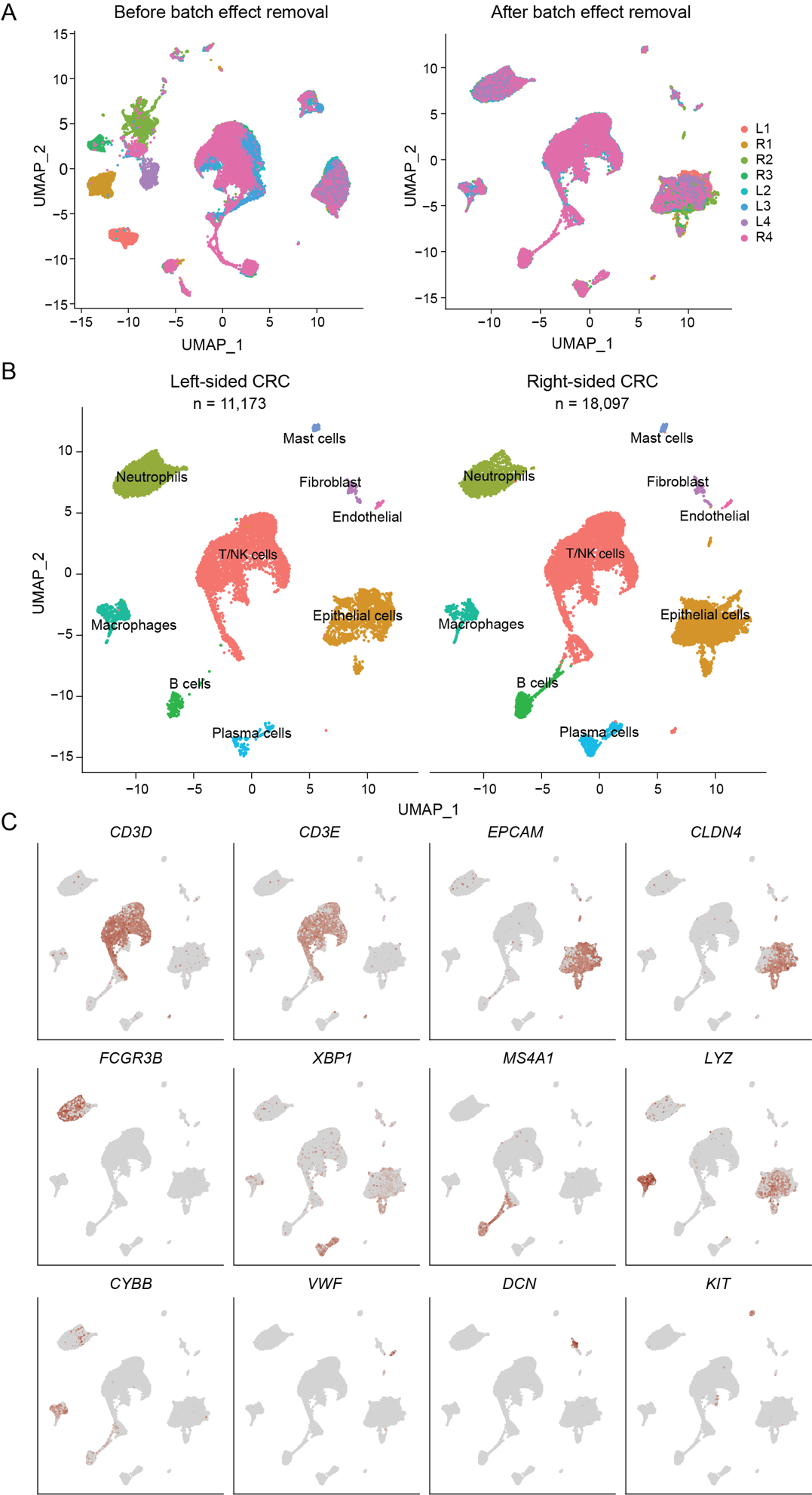
**

**Figure S1. Single-cell transcriptomic atlas of left-sided and right-sided CRC.** A. UMAP by samples before and after batch effect removal. B. UMAP by cell clusters of left-sided and right-sided CRC. C. Expression levels of representative known markers illustrated in UMAP plots.

**
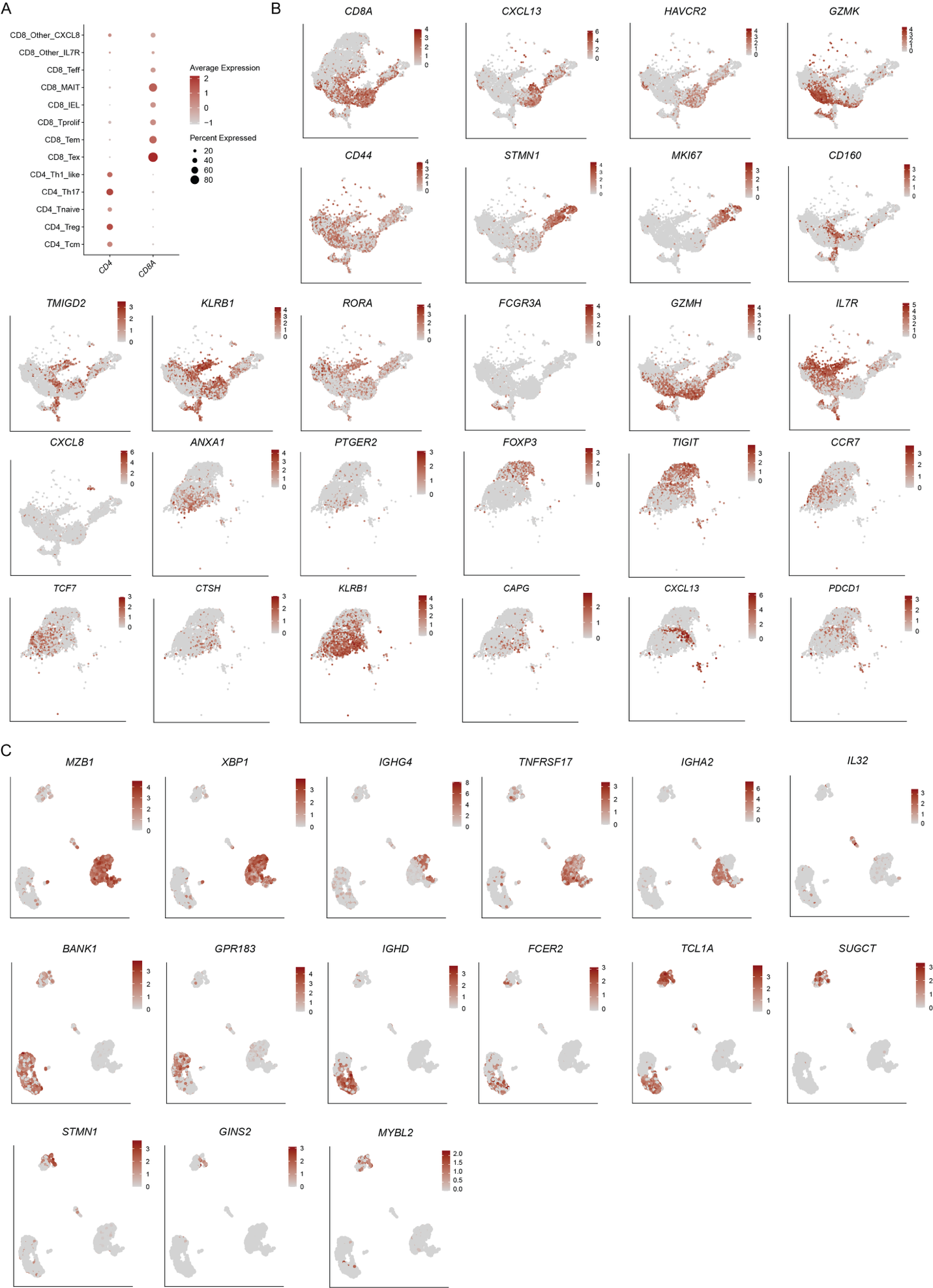
**

**Figure S2. T cell and B cell subclusters.** A. Dot plot of *CD4* and *CD8A* expression in subclusters. B. Expression levels of representative known markers in CD4^+^ and CD8^+^ T cells are illustrated in UMAP plots. C. Expression levels of representative known markers in B cells are illustrated in UMAP plots.


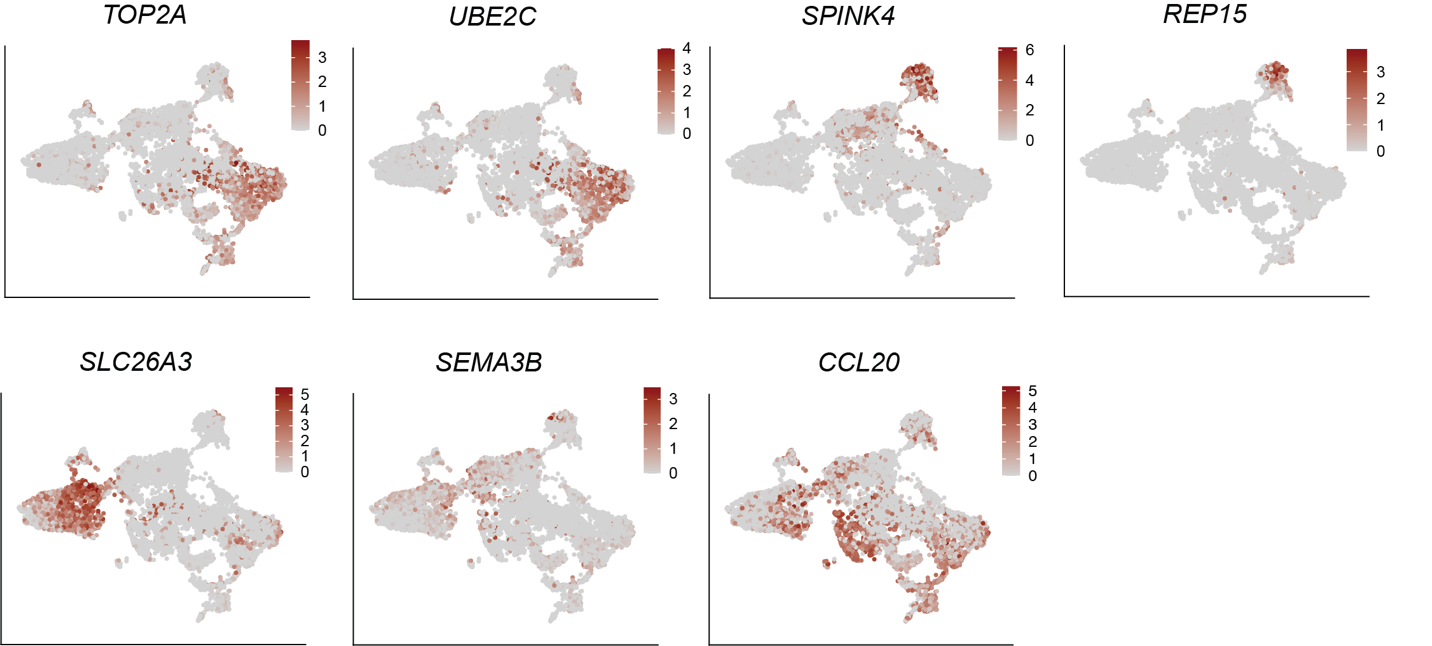


**Figure S3. Epithelial subclusters.** Expression levels of representative known markers illustrated in UMAP plots.


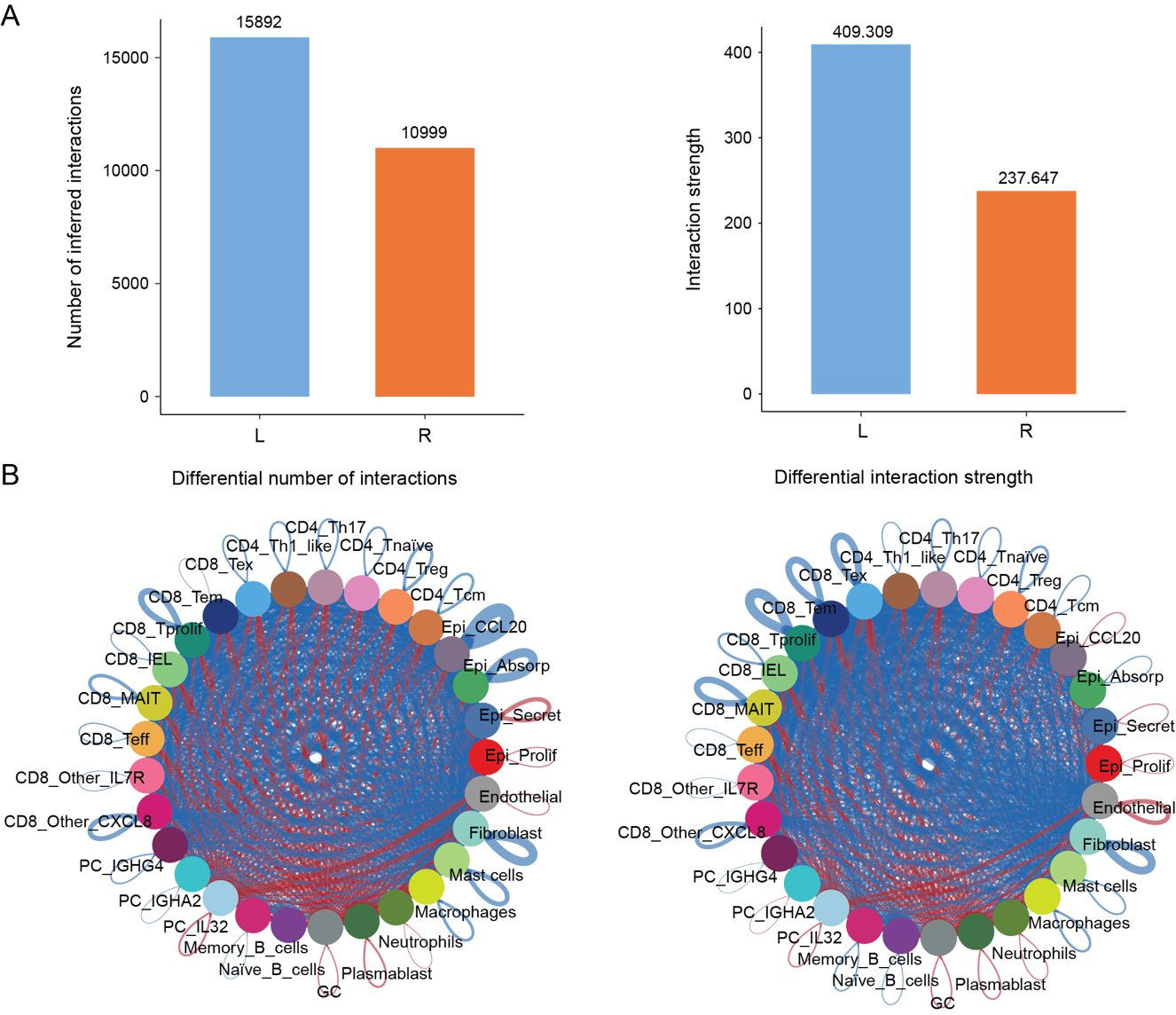


**Figure S4. Intercellular communications between sub-clusters.** A. Number of interactions and strength of interactions in left-sided and right-sided CRC. B. Circo plots showing the differential number of interactions and the differential strength of interactions. Blue, enriched in the left-sided CRC. Red, enriched in right-sided CRC.


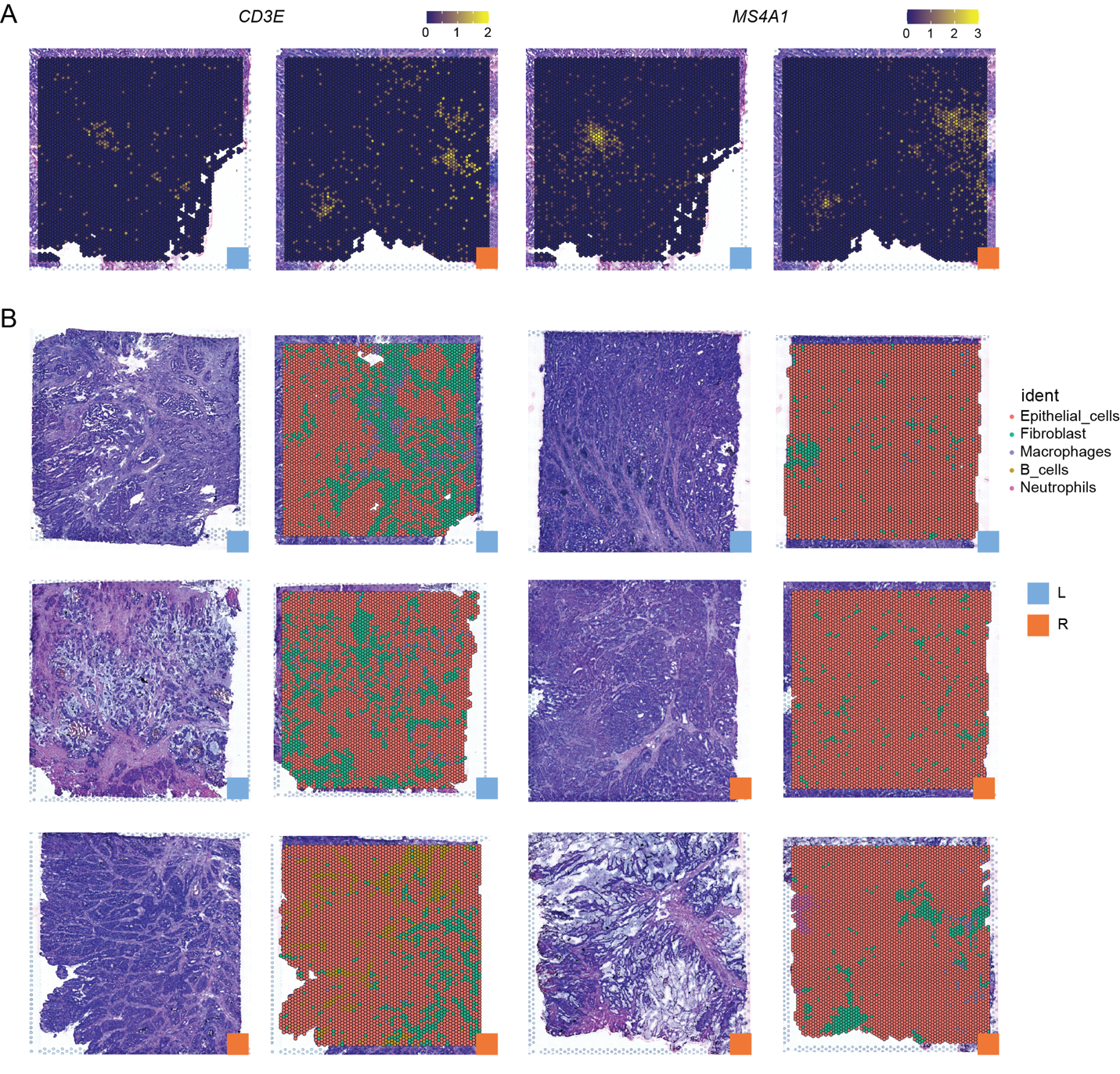


**Figure S5. Spatial transcriptomics revealed gene expression and clustering.** A. Spatial feature plots of gene expression of *CD3E* and *MS4A1.* B. Clustering of ST spots in left- and right-sided CRC nests.


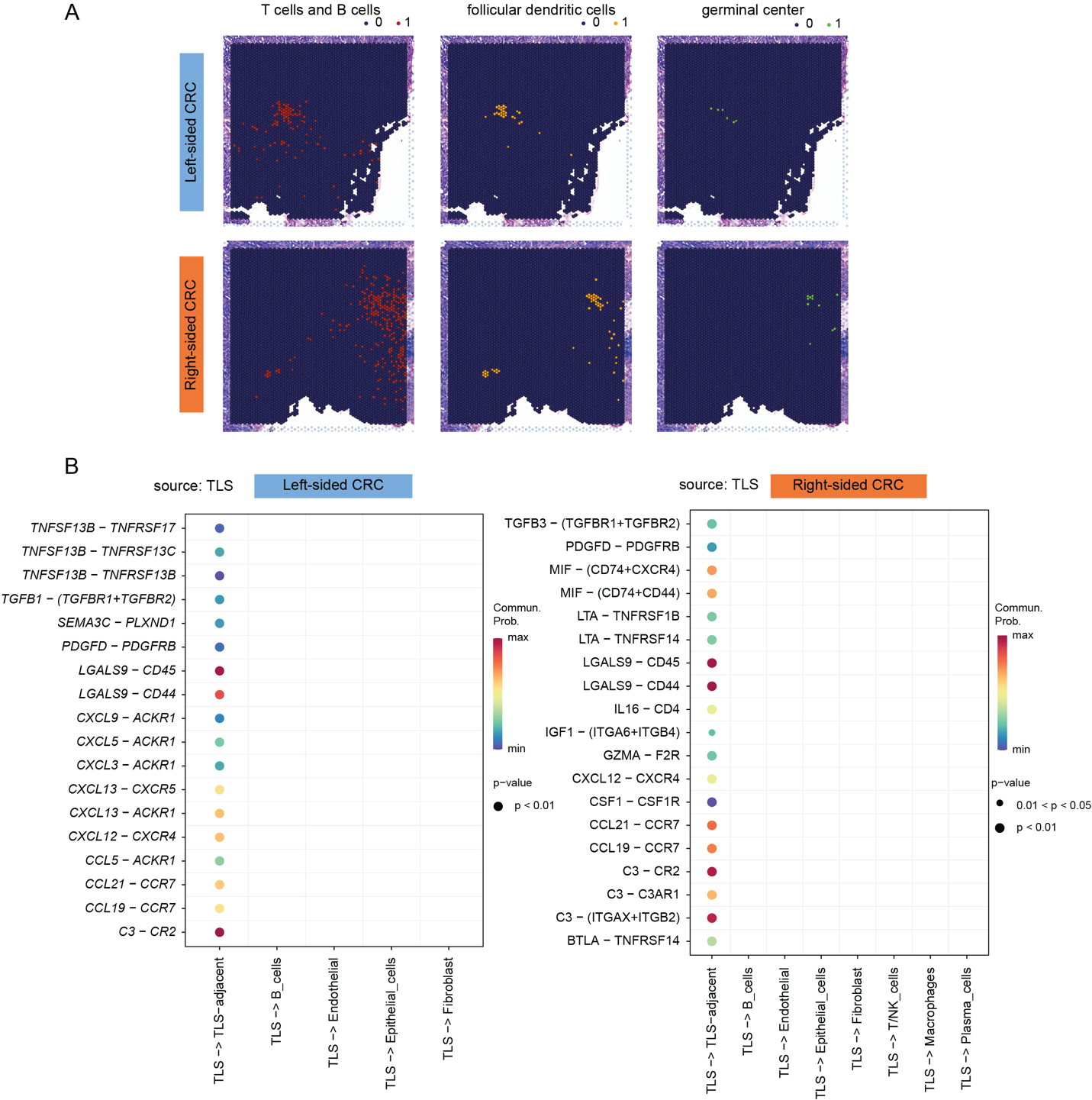


**Figure S6. Spatial transcriptomics revealing identification of cell types and interactions.** A. Identification of T/B cells, follicular dendrite cells and germinal centers based on add module scores. B. Bubble heatmap showing the communication probability between clusters for ligand-receptor pairs using the TLS cluster as source.

**Supplemental Table 1. Patient information.**

| **ID** | **Gender** | **Age** | **Tumor Location** | **Differentiation** | **Mucinous Adenocarc-inoma** | **Pathological Stage** | **Nerve and Vessel Invasion** | **Tumor Budding** | **MSI** | ***BRAF*** | **Chemoradiot-herapy Before Surgery** | **Survival (as of July 22^th^, 2024)** |
| --- | --- | --- | --- | --- | --- | --- | --- | --- | --- | --- | --- | --- |
| L1 | Male | 77 | Sigmoid colon | Low | Yes | Stage Ⅱ  (T4N0M0) | Yes | BD3 | pMMR | Wild type | None | Alive  (30 months) |
| R1 | Female | 76 | Cecum | Low | Yes | Stage Ⅲ  (T3N1M0) | No | - | dMMR | Wild type | None | Alive  (30 months) |
| R2 | Male | 61 | Ascending colon | Moderate | No | Stage Ⅲ  (T3N2MO) | Yes | BD3 | pMMR | Wild type | None | Alive  (29 months) |
| R3 | Male | 66 | Ascending colon | Moderate | Yes (Focal) | Stage Ⅱ  (T4N0M0) | Yes | BD1 | pMMR | Wild type | None | Alive  (29 months) |
| L2 | Male | 34 | Rectum | Moderate | No | Stage Ⅲ  (T2N1MO) | No | - | pMMR | Wild type | None | Alive  (16 months) |
| L3 | Female | 73 | Sigmoid colon | Moderate | Yes | Stage Ⅱ  (T3N0M0) | No | BD2 | pMMR | Wild type | None | Alive  (28 months) |
| L4 | Male | 56 | Rectum | Moderate | No | Stage Ⅱ  (T3N0M0) | No | BD1 | pMMR | Wild type | None | Alive  (14 months) |
| R4 | Male | 68 | Ascending colon | Moderate | No | Stage Ⅱ  (T3N0M0) | No | BD2 | pMMR | Wild type | None | Alive  (12 months) |
